# Role of IgM/ IgG Ratio in Distinguishing Primary and Secondary Dengue Viral Infections: A Cross-Sectional Study

**DOI:** 10.7759/cureus.66714

**Published:** 2024-08-12

**Authors:** Charu Kalra, Garima Mittal, Priyanka Gupta, Rajiv Kumar Agarwal, Sohaib Ahmad

**Affiliations:** 1 Microbilogy, Himalayan Institute of Medical Sciences, Swami Rama Himalayan University, Dehradun, IND; 2 Microbiology, Himalayan Institute of Medical Sciences, Swami Rama Himalayan University, Dehradun, IND; 3 Microbiology, Gautam Buddha Chikitsa Mahavidyalaya, Subharti University, Dehradun, IND; 4 Internal Medicine, Himalayan Institute of Medical Sciences, Swami Rama Himalayan University, Dehradun, IND

**Keywords:** elisa, pcr, igm :igg ratio, secondary dengue, primary dengue

## Abstract

Objectives

In recent years, Uttarakhand, a state in North India has become one of the prime spots for tourism all over the world. Thereby, a tremendous increase in the epidemics of dengue infection has been observed recently. Secondary dengue causes more severe disease in comparison with primary, thus to differentiate the two is very crucial. We aim to find out the cut-off values of the IgM:IgG ratio for early detection of secondary dengue which could further help clinicians to prevent the complications.

Methods

A cross-sectional study was conducted over one year involving around 936 suspected cases of dengue. Samples were tested using the commercially available capture enzyme linked Immunosorbent assay (ELISA) method for IgM and IgG. Real-time and nested polymerase chain reaction (PCR) tests were also done to find out the prevalent serotype. IgM:IgG ratio was evaluated by using receiver operating characteristic curve analysis for the differentiation of primary and secondary dengue.

Results

Among the total 91 serologically confirmed dengue patients, forty-seven (51.6%) were found to be primary, and forty-four (48.4%) were secondary dengue infections with male preponderance. Using the WHO diagnostic criteria, patients with dengue fever (DF) without warning signs added up to 51.6%, with warning signs 42.9% and severe dengue 5.5% of the total cases. The cut-off ratio of IgM:IgG ratio = 1.59 found the best discrimination between primary and secondary infection. Forty out of ninety-one (44%) patients exhibited ratios of > 1.59 whereas the rest fifty-one (56%) exhibited ratios of < 1.59. Dengue virus - 2 (DENV- 2) was found to be the most prevalent serotype.

Conclusion

Our study recommends the cut-off values for IgM:IgG ratio as 1.59. Therefore it is hoped that this will guide the clinicians to early distinguish between primary and secondary dengue. Furthermore, it can reduce morbidity and mortality because of dengue infections in the future.

## Introduction

Dengue is a disease with varying clinical presentations and often with unpredictable clinical evolution and outcome. Dengue has been ranked highest among the re-emerging, serious arboviral infectious diseases transmitted by Aedes aegypti and Aedes albopictus mosquitoes [1]. “Dengue Virus”, is a single-stranded RNA virus that belongs to the family of Flaviviridae and has four serologically related but genetically distinctive virus serotypes, namely, dengue virus (DENV)-1, DENV-2, DENV-3, and DENV-4 all of them capable of causing serious illness [2]. A fifth serotype has also been identified recently The discovery of DENV-5 epitomizes an autonomous cross-species transmission of sylvatic form of dengue to humans [3].

The infection with any one particular serotype is known to offer protection against re-infection with the same serotype, but no cross-protection against the other serotypes. These four serotypes are genetically similar and share approximately 65% of their genomes. Each serotype generates a unique host immune response to the infection [2].

Primary dengue infection occurs when a previously non-immunized person is infected with any one serotype and subsequent infection with another serotype (secondary infection) or multiple infections with different serotypes more often leads to severe complications like dengue hemorrhagic fever (DHF) and dengue shock syndrome (DSS) [4,5]. Dengue as a whole is endemic across the whole of India and different strains have been responsible for dengue infections in different regions at different times [6].

In recent years, India has reported an increasing number of dengue cases, with some years experiencing over 100,000 reported cases. The World Health Organization (WHO) estimates that there are about 100-400 million dengue infections each year globally [7,8].

Both adults and older children in the dengue-affected areas are likely to have been exposed to the dengue virus in the past, and this makes them more prone to secondary infections [9]. The lack of tools to assess the immune status of patients to mark out the progress of the disease often leads to an enormous number of preventable and costly hospitalizations. Numerous virologic and immunological parameters have been considered useful in delineating the pathogenesis of dengue which however varies between individuals and with primary and secondary infections [10,11]. Differentiating between primary and secondary dengue infections is important from a prognostic and epidemiological point of view.

IgM antibodies are demonstrable in the serum of the patients from three to five days after the onset of illness keep on rising for nearly two weeks and persist for nearly 179 and 139 days in primary and secondary infection, respectively [12]. On the contrary, in the acute phase of secondary dengue infections, high levels of cross-reactive IgG antibodies are detected with prior or simultaneous IgM response [13]. The rapid increase in IgG levels in the early days of illness during secondary infection is indicative of dengue when the IgM and IgG ratios are calculated [12-15].

It has been suggested that methods that discriminate primary and secondary DENV infection may be important from a prognostic point of view. Haem-agglutination inhibition (HAI) assay was earlier the gold standard test to differentiate between primary dengue and secondary dengue infections. However, due to various practical limitations and requirements of paired sera, early diagnosis of the disease cannot be made. Nowadays, IgM and IgG capture enzyme linked immunosorbent assay (ELISA) has replaced HAI assay and has become the most powerful assay for dengue diagnosis. They are being widely used for diagnosis due to the possibility of automation, simplicity, high sensitivity, and speciﬁcity [16-18].

The present study was undertaken to determine the best cut-off point of IgM:IgG ratio in acute phase samples in this region so as to differentiate primary and secondary dengue infection and to determine the prevalent serotype of DENV in this area.

## Materials and methods

A cross-sectional study was conducted in the Department of Microbiology in a tertiary care teaching hospital over 12 months from January 2017 to December 2017. Subjects were recruited from patients presenting in inpatient and outpatient departments of the hospital with a history of acute febrile illness and written informed consent was taken. All the relevant clinical, demographical, laboratory, and serological data were collected on the day the patient attended the health facility. Ethical clearance was obtained from the Institutional Ethical Committee vide letter no. SRHU/HIMS/ETHICS/2018/94 dated 16/07/2018. Patients of all age groups and either sex attending tertiary care hospital with a fever history of two to seven days duration and ≥ 2 of symptoms including headache, retro-orbital pain, myalgia, arthralgia, rash, and hemorrhagic manifestation were included in the study [19], whereas patients with fever more than seven days duration, any history of urinary tract infection, evidence of pneumonia, abscess or other obvious causes of fever were excluded.

Blood samples from 936 patients with acute febrile illness as per the inclusion criteria of the study were collected and subjected to microbiological tests. Five milliliters of blood was collected aseptically, and serum was separated by centrifuging the vacutainers at 1,600Xg for 10 minutes. Out of the total 936 samples, 234 tested positive for dengue either by NS1 antigen or IgM antibody testing. Ninety-one random samples (based on a computer-generated random number table) were stored in cryovials at -20˚C for later use in IgG serology and polymerase chain reaction (PCR), as all the samples could not be processed because of feasibility and financial issues. Standard diagnostics Bioline (SD BIOLINE) Dengue Duo NS1 + Ab combo kit was used to determine NS1, IgM, and IgG results (sensitivity 92.4%, specificity 98.4% of NS1 and sensitivity 94.2%, specificity 96.4% of Ab). IgM and IgG index values are calculated by using Panbio Dengue IgM (sensitivity 94.7%, specificity 100%) and IgG capture ELISA (sensitivity 85.7%, specificity 100%) respectively. All the tests were performed, and the results were calculated and interpreted as per the manufacturer’s instructions.

Criteria for primary and secondary infection

Primary DENV infection was defined as either NS1 Ag+ IgM- IgG- or IgM+ IgG-. Whereas secondary DENV infection was defined as an IgM- IgG+ or IgM+ IgG+.

Reverse transcription polymerase chain reaction (RT-PCR)

The viral RNA was extracted from the 50 serum samples using the commercial QI Amp Viral RNA Mini kit (QIAGEN, Germany) according to the manufacturer’s kit instructions. Real-time PCR was carried out and two-step nested PCR was done on samples with high viral load of dengue virus for serotype detection. Extracted viral nucleic acid was first converted to cDNA by reverse transcription and then amplified in a thermocycler. The primer sequences described by Lanciotti et al. were used [20]. Amplified products were separated by agarose gel electrophoresis through 1.6% agarose gel in Tris-acetate-EDTA (TAE) buffer. Electrophoresis was carried out at 100-150 Volts for 45 min or until the bands were resolved. Gel was screened under UV light on an electronic UV transilluminator gel documentation system for the presence of 511 bp bands of DNA. After the first round, only those samples were processed for serotyping which showed 511 bp bands. The forward primer and the serotype-specific reverse primers primer TS1, TS2, TS3, and TS4 for DENV-1, DENV-2, DENV-3 and DENV-4 respectively targeting C-pre M gene junction were used for detection. Amplification products were separated by gel electrophoresis as described above. With the help of a gel documentation system, the serotype-specific bands of each dengue serotype were visualized. Bands specific for DENV-1, DENV-2, DENV-3 and DENV-4 of respective base pairs482bp, 119bp, 290bp and 392bp were tested. To avoid the risk of false positives, both positive and negative controls were included in the assay.

Data management & statistical analysis

Recorded data was analyzed using SPSS (IBM Corp. Released 2011. IBM SPSS Statistics for Windows, Version 20.0. Armonk, NY: IBM Corp). The demographic profile, clinical features, and laboratory investigations were described by descriptive statistics. The categorical variables were compared by Pearson Chi-Square and Fisher exact probability test. The independent sample unpaired t-tests were used to assess the statistical difference between the continuous variables conforming to normal distribution whereas the distribution which was skewed was compared using the Mann-Whitney U-test. Sensitivity, specificity, likelihood ratio, accuracy, and cut-off point for IgM:IgG ratio were determined by the receiver operator characteristic (ROC) curve.

## Results

Among the 91 serologically confirmed dengue cases, 51.6 % (47) were of primary dengue infection, and 48.4% (44) were of secondary dengue infection. The mean age and sex distribution among primary and secondary dengue is shown in Table 1.

**Table 1:** Comparison of demographic, clinical, and laboratory parameters in primary and secondary dengue infection (n=91) AST: aspartate transaminase, ALT: alanine transaminase. Note: P value < 0.05 is considered to be significant

Parameters		Primary dengue n = 47	Secondary dengue n = 44	Total n = 91	P value
Male (M)	n (%)	31 (66)	31 (70.5)	62 (68.1)	0.645
Female (F)	n (%)	16 (34)	13 (29.5)	29 (31.9)	
Age (years)	Mean ± SD (Median)	29.79 (18.33) (27)	35.45 (19.99) (32.50)	32.53 (19.26) (29)	0.181
Headache	n (%)	41 (87.23)	31 (70.45)	72 (79.12)	< 0.05
Lethargy	n (%)	32 (68.1)	38 (86.36)	70 (76.92)	< 0.05
Fever > 99.5 º F	n (%)	31 (65.95)	29 (65.90)	60 (65.93)	0.996
Aches & Pains	n (%)	28 (59.6)	27 (61.4)	55 (60.4)	0.862
Gastrointestinal symptoms	n (%)	21 (44.7)	21 (47.7)	42 (46.2)	0.771
Hemorrhagic manifestation	n (%)	19 (40.42)	27 (61.36)	46 (50.54)	< 0.05
Retro-orbital pain	n (%)	11 (23.4)	11 (25)	22 (24.2)	0.859
Hemoglobin (g /dl)	Mean ± SD (Median)	13.2 ±2.94 13.9	13.5 ±3.34 (14.1)	13.39 ±3.12 (14.03)	0.606
Total leucocyte count (x10^3^/mm^3^)	Mean ± SD (Median)	9.21 ± 6.57 (7.37)	9.34 ±5.59 (7.61)	9.28 ±6.08 (7.43)	0.589
Leucopenia (x10^3^/ mm^3^)	Mean ± SD (Median)	2.89 ± 0.749 (2.79)	3.72 ±0.552 (4)	3.33 ±0.76 (3.39)	< 0.05
Platelet count (x10^3^/ mm^3^)	Mean ± SD (Median)	54.1 ± 52.3 (30)	53.9 ±63.8 (31)	54.1 ±57.8 (30)	0.802
AST / ALT	Mean ± SD (Median)	2.077 ± 1.17 (1.72)	2.27 ±1.35 (2.06)	2.17 ±1.26 (1.8)	0.341
Alkaline Phosphatase (ALP IU/L)	Mean ± SD (Median)	132.78 ±110.4 (92)	172.57 ±124.7 (145)	152.4 ±118.6 (108)	0.083
A/G ratio	Mean ± SD (Median)	1.08 ±0.421 (1)	1.03 ±0.280 (0.97)	1.05 ±0.356 (1)	0.616
Blood urea nitrogen (mg/dl)	Mean ± SD (Median)	13.2 ±13.7 (9.2)	13.9 ±9.43 (10.3)	13.5 ±11.77 (9.85)	0.266
IgM Index	Mean ± SD (Median)	2.21 ±0.78 (2.06)	2.05 ±0.875 (2.08)	2.13 ±0.825 (2.07)	0.349
IgG Index	Mean ± SD (Median)	0.906 ± 0.462 (1)	4.66 ±1.917 (3.63)	2.72 ±2.329 (1.775)	< 0.0005
IgM:IgG Index	Mean ± SD (Median)	2.99 ± 1.64 (2)	0.532 ± 0 .346 (0.410)	1.806±1.722 (1.17)	< .0005

The study group presented commonly with headache (79.12%), lethargy (76.92 %), fever (65.93%) and other symptoms. Headache, lethargy, and hemorrhagic manifestations were significantly associated with the disease (P value < 0.05). 

Clinical outcomes in both groups have been compared in (Table 2).

**Table 2:** Comparison of clinical outcome in primary and secondary dengue infection (n=91) Note: P value < 0.05 is considered to be significant

Parameters		Primary	Secondary	Total	P value
Morbidity					
Hospitalization days	Mean(SD) (Median)	4.83±2.77 (4)	4.82 ±2.43 (4)	4.82±2.60 (4)	0.793
Platelet recovery days	Mean(SD) (Median)	1.81 ±0.821 (2)	2.13 ±0.957 (2)	1.97 ±0.897 (2)	0.193
Acute liver failure	n (%)	17 (36.4)	28 (63.6)	45 (49.5)	0.016
Acute renal failure	n (%)	6 (12.8)	13 (29.5)	19 (20.9)	0.087
Multi-organ Dysfunction	n (%)	1 (2.1)	6 (13.6)	7 (7.7)	0.096
Shock	n (%)	2 (4.3)	3 (6.8)	5 (5.5)	0.054
Seizures	n (%)	1 (2.1)	-	1 (1.1)	0.331
Encephalopathy	n (%)	-	1 (2.3)	1 (1.1)	0.299
Intensive care	n (%)	4 (8.5)	6 (13.6)	10 (11)	0.435
Case fatality rate		-	2.27	1.09	-

**Figure 1:**
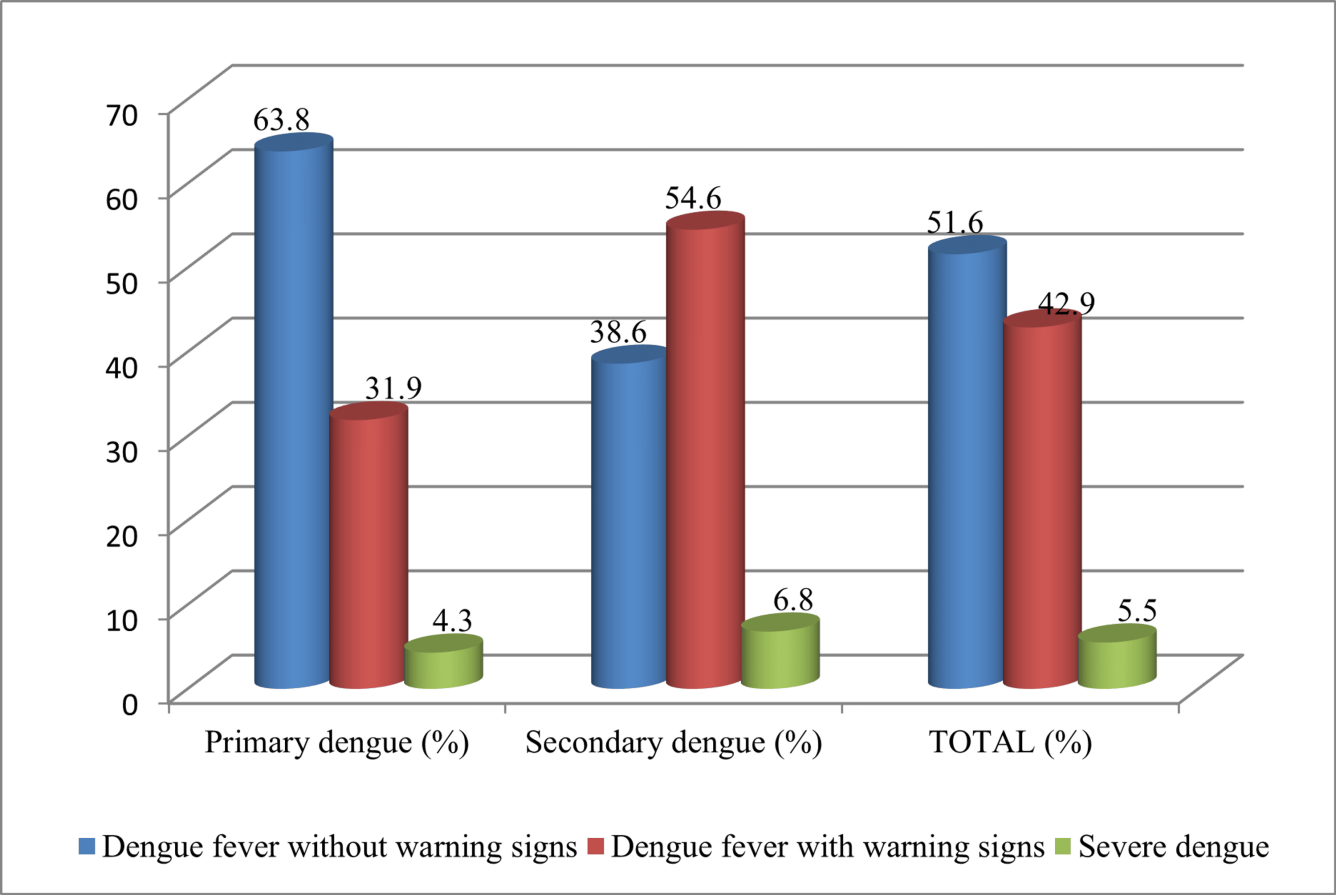
Categorization of primary and secondary dengue cases as per WHO classification (n=91)

As per the WHO 2009 classification patients were grouped into: dengue fever without warning signs, dengue fever with warning signs, and severe dengue (Figure 1).

The best cut-off of IgM:IgG ratio was found to be 1.59 with a sensitivity of 85.11 %, specificity of 100 %, accuracy level of 92.3%, positive (-), and negative likelihood ratio (0.15) as compared with others (Table 3).

**Table 3:** Analytical values of various cut-off points of IgM:IgG ratio in differentiating primary and secondary infections PPV: positive predictive value, NPV: negative predictive value, PLR: positive likelihood ratio, NLR: negative likelihood ratio.

IgM:IgG Ratio	Sensitivity (%)	Specificity (%)	PPV (%)	NPV (%)	Accuracy (%)	PLR	NLR
1.09	93.62	90.91	91.67	93.02	92.31	10.3	.07
1.16	91.49	93.18	93.48	91.11	92.3	13.42	.09
1.21	87.23	93.18	93.18	87.23	90.11	12.79	.14
1.40	85.11	97.73	97.56	86.00	91.21	37.45	.15
1.59	85.11	100	100	86.27	92.31	-	.15
1.77	82.98	100	100	84.62	91.21	-	.17
1.87	78.71	100	100	81.48	89.01	-	.21

**Figure 2:**
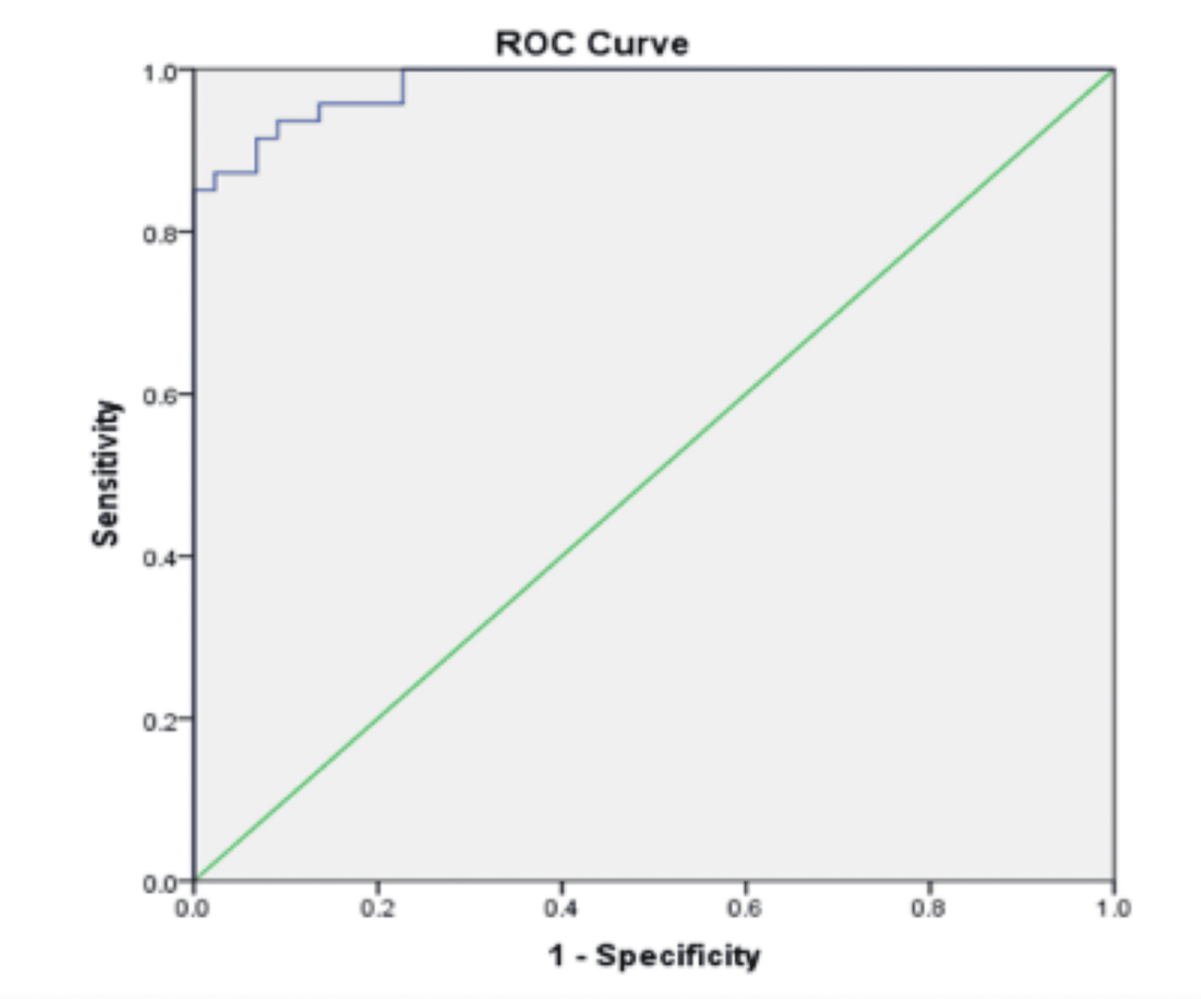
Receiver operator characteristic (ROC) curve for determining IgM:IgG cutoff

It had shown good performance by the ROC curve as the area under the ROC curve was 0.982 (Figure 2). With the best cut-off ratio of 1.59, 44% (40 cases) were thus classified as primary dengue and 56% (51 cases) as secondary dengue.

Forty-five out of 50 samples came positive by RT-PCR leading to a positivity rate of 90%. All these samples were positive by ELISA. In the present study, DENV-2 was found to be the most prevalent serotype detected both in primary and secondary dengue patients than DENV-1 and was isolated in patients who have been classified as dengue fever with warning signs and severe dengue (Table 4).

**Table 4:** Dengue virus serotype associated with primary and secondary dengue infection and serotype distribution according to severity of dengue infection * 3 samples of primary cases were degraded; ** 1 sample of secondary case was degraded.

	No of samples serotyped	DENV-1 n (%)	DENV-2 n (%)
Primary Dengue Infection	*22	8 (36.36)	11 (50)
Secondary Dengue Infection	**13	5 (38.46)	(53.84)
WHO definition			
Dengue fever without warning signs	21	11 (52.38)	7 (33.33)
Dengue fever with warning signs	11	2 (18.18)	9 (81.8)
Severe dengue	3	--	2(66.6)

A comparison between IgM:IgG cut-off ratios for differentiating primary and secondary infections in various studies is mentioned in (Table 5).

**Table 5:** IgM:IgG cut-off ratios for differentiating primary and secondary infections in various studies

Study	Country	IgM:IgG ratio
Innis et al. [16]	Bangkok, Thailand	1.78
Chanama et al. [35]	Nonthaburi, Thailand	1.8
Kuno et al. [17]	Puerto Rico	1.4
Prince et al. [36]	California, USA	1.32
Shu et al. [18]	Taiwan, Republic of China	1.2
Cucunawangsih et al. [37]	Indonesia	1.14
Our study	India	1.59

## Discussion

In the Indian subcontinent, the trajectory of dengue fever has undergone a discrete change over decades. It has changed in terms of the severity of illness with different prevailing strains and different geographical locations being affected. Its incidence has increased significantly [7].

Dengue is endemic in more than 100 countries and still, it has been categorized as a “neglected tropical disease” [21]. The delay in diagnosis as well as appropriate management is substantially associated with morbidity and mortality. Out of 91 serologically confirmed dengue cases, 51.6 % were of primary dengue infection and 48.4% were of secondary dengue infection. The primary to secondary dengue infection ratio was 1.07:1.59% primary dengue, 41% secondary dengue infection, and a primary: secondary ratio of 1.4:1 has been reported [22], which correlates well with the present study. While another study has observed 34.3% primary dengue and 65.7% secondary dengue [23].

In our study, the number of males (68.1 %) with dengue illness was considerably higher than females (31.9 %); overall male; female ratio was 2.13:1. Comparable pattern of male predominance has also been found in other studies all across the globe [24-26]. However, equitable sex distribution has also been documented [27,28]. The main reason for male preponderance in the present study could be that in our Indian society males are considered to be the earning members of the family and spend more time outdoors to make their livelihood, hence are more exposed to insects. Also, differential seeking behavior of medical care for females in whom mild illnesses are generally neglected could account for the same. The highest prevalence of dengue infection was observed in the economically productive age group. In the present study, the mean age group in primary dengue infection was 29.79 ± 18.3 years, whereas in secondary dengue infection, the mean age group was 35.45 ± 19.9 years. An adult predominance in dengue fever with the mean age of patients being 27.5±11.7 years and 30.7±14.4 years in primary dengue and secondary dengue fever were observed respectively [29]. Fever in the hospital after admission was documented in 65.95 % of patients; however, 100% of patients had given a history of fever for four to six days prior to their hospital visit. Those who were not presenting with fever had visited the hospital for consultation for the following complaints: headache aches and pains, lethargy, gastrointestinal symptoms, and hemorrhagic manifestations. These findings are in congruence with the report in this same institute in 2013 in which 66.3% of patients had fever on presentation in the hospital [30]. On stratified analysis, patients with primary dengue were significantly associated with headache, however, patients with secondary dengue infection were significantly associated with lethargy and hemorrhagic manifestation (P value < 0.05). Our findings of common symptoms are in concordance with the findings of others [29].

Of the common morbidities found in our study namely acute liver failure (49.5%), acute renal failure (AKI) (20.9%), multi-organ dysfunction (MODS) (7.7%), shock (5.5 %), seizures (1.1%), encephalopathy (1.1%) and intensive care requirement (11%), the first three were found to be significantly allied with the disease (P value < 0.05). Mallhi et al. in 2015 had reported similar findings except for MODS which was found to be at least three times less in our work than their study [31]. MODS occurs as a virus that can replicate in hepatocytes, glomeruli, pneumocytes, cardiac fibers, macrophages, and endothelial cells, and acute renal impairment could be due to direct virus injury or due to immune complex deposition in the glomerulus [31]. A study conducted in the same institute made observations on these parameters and reported a lower mean duration of hospitalization and mean platelet recovery time, shock in 3.4%, and requirement of intensive care in 5.1% in dengue illness. However, a higher rate of seizures was present in 6.8%of dengue patients as compared to our study [32].

As per the WHO 2009 classification, in the present study 51.6% cases of DF without warning signs, 42.9% of DF with warning signs, and 5.5% cases of severe dengue were observed. The difference in proportions between primary and secondary dengue came out to be statistically significant in the first two classes (P values < 0.05). Comparable findings have been reported earlier [33]. They have also opined that the new WHO classification (2009) is more competent in triaging severe dengue cases which they felt was missed in 1997 [33].

In a comparative study in Uttarakhand in 2015 and 2016, the case fatality ratio was reported as 1.25% and 2.06% respectively [34]. These findings are conducive to the results of the present study where the case fatality rate is 1.09 in severe dengue cases.

Antibody capture ELISA was performed on serum samples from dengue patients collected on various days of infection. IgM antibodies were detected in 51.64% of the samples and IgG in 11% whereas both IgM & IgG were detected in 37.36% of patients. These figures are slightly different than the findings of other workers. The mean IgM index, IgG index, and IgM:IgG index were also calculated for primary and secondary infection and a significantly higher association was seen in the IgG index and IgM:IgG index with dengue infection.

Various workers have calculated the IgM:IgG cut-off ratio to be in the range of 1.2 to 2.0 depending upon the various diagnostic assays and interpretation protocol (Table 5). The workers in various studies have reported this cut-off ratio to be as follows: 1.78 [16], 1.4 [17], 1.2 [18], 1.8 [35], 1.32 [36] and 1.14 [37]. The possible reasons for different ratios in these studies could be the different settings and sero-epidemiology. In our study, we have found IgM:IgG ratio of 1.59 as the best cut-off for differentiating acute primary and acute secondary dengue infection in this region.

In the present study, DENV-2 was found to be the most prevalent serotype detected both in primary and secondary dengue patients and was found associated with a severe form of dengue illness whereas DENV-1 was isolated mainly from patients with dengue fever without warning signs. DENV-2 is among the most virulent serotypes of dengue infection and this could be the reason that in our study differences in clinical profile, lab parameters, days of hospitalization, and platelet concentrate transfusion rate were not significantly seen in primary and secondary dengue patients. Soo et al. [38], in their meta-analysis study, have mentioned that the dengue serotypes involved in causing dengue infection and the interval between the primary and secondary infections affected the severity of dengue infection. So, these factors must be taken into consideration while doing the clinical assessment of the severity of dengue patients. DENV-2 and DENV-4 are more commonly associated with secondary DENV infection. Therefore, patients who have been exposed to dengue virus previously must be cautious during outbreaks of DENV-2 and DENV-4 infections [38].

Limitations

This study had a few limitations, including a small sample size. Future studies should consider a larger sample size to enhance the reliability of the findings. Additionally, due to financial constraints, we were unable to perform RT-PCR testing on all study subjects. With appropriate financial support, these limitations can be addressed in future research.

## Conclusions

Based on this thorough study, a first of its kind in this region of Uttarakhand, the cut-off value for IgM:IgG ratio is recommended as 1.59. It is hoped that this will guide clinicians to diagnose dengue more accurately and this would go a long way in reducing the morbidity, mortality, and economic burden of this very prevalent, obnoxious, and possibly fatal disease, especially in the secondary dengue cases.
